# Carbon-Coatings Improve Performance of Li-Ion Battery

**DOI:** 10.3390/nano12111936

**Published:** 2022-06-06

**Authors:** Ziling Chen, Qian Zhang, Qijie Liang

**Affiliations:** 1 Songshan Lake Materials Laboratory, University Innovation Park, Songshan Lake, Dongguan 523808, Guangdong, China; chenziling@sslab.org.cn; 2 School of Materials, Shenzhen Campus of Sun Yat-sen University, No. 66, Gongchang Road, Guangming District, Shenzhen 518107, China

**Keywords:** lithium-ion battery, carbon coating, specific capacity, cycling stability

## Abstract

The development of lithium-ion batteries largely relies on the cathode and anode materials. In particular, the optimization of cathode materials plays an extremely important role in improving the performance of lithium-ion batteries, such as specific capacity or cycling stability. Carbon coating modifying the surface of cathode materials is regarded as an effective strategy that meets the demand of Lithium-ion battery cathodes. This work mainly reviews the modification mechanism and method of carbon coating, and summarizes the recent progress of carbon coating on some typical cathode materials (LiFePO_4_, LiMn_2_O_4_, LiCoO_2_, NCA (LiNiCoAlO_2_) and NCM (LiNiMnCoO_2_)). In addition, the limitations of the carbon coating on the cathode are also introduced. Suggestions on improving the effectiveness of carbon coating for future study are also presented.

## 1. Introduction

Lithium-ion batteries (LIB) are well known as the most promising candidate in the electrochemical energy storage system and power source, due to the excellent features of light weight, high power and energy density, high current discharge, and long service lifetime [1,2]. It is considered by scientists and governments because of the extremely broad application in the portable consumer electronics and automobile market [3]. To meet the requirement of energy storage, decreasing the energy consumption [4,5] and developing the next generation, LIB becomes increasingly important [6,7].

Although the LIB has been widely applied in some fields and markets, the issues that affect the performance of LIB still have not been completely solved [8]. Particularly, the energy density of LIB still needs to be improved. It remains a challenge to find electrode couples with both high specific capacity and high cycling stability. At the same time, the types and performance of cathode materials are directly related to specific capacity and rate performance of LIB [9,10]. On the other hand, the cycling stability is affected by the degradation mechanism of LIB during operation. The reaction at the electrode–electrolyte interface results in poor cell lifetimes. Therefore, developing the electrode materials of LIB is the primary step.

The cathode is the most expensive and heaviest component in the LIB compared with the anode. Therefore, the cathode currently limits the specific capacity of LIB and mainly determines the cost of LIB [11]. Optimizing the cathode is of great importance for performance improvement of the LIB. At present, the common cathode materials could be classified in three types: (1) olivine structure (such as LiFePO_4_), (2) spinels structure (such as LiMn_2_O_4_) and (3) layered oxide structure (such as LiCoO_2_, LiNiO_2_, NCA (LiNiCoAlO_2_) and NCM (LiNiMnCoO_2_)) [12]. LiCoO_2_ with a two-dimensional layered structure has the advantages of simple preparation process, high specific capacity and good cycling performance. LiMn_2_O_4_ has a three-dimensional tunnel structure, which is more suitable for the lithiation and de-lithiation reaction, and hence it has decent power capacity and thermal stability. However, it suffers from structure instability, resulting in the easy loss of LIB capacity. By contrast, LiFePO_4_ exhibits higher practical capacity, better structural stability and good cycle life. Nevertheless, the problems of LiFePO_4_ include low energy density and poor performance at low temperature. Recently, various metals such as nickel, manganese and aluminum have been combined with cobaltates, including NCA and NCM. However, these types of cathode materials still suffer from similar problems, which limit their further development [13]. To overcome issues of low specific capacity and poor cycling stability in the cathode materials, it has been reported that surface coating is an effective and economical method [14]. Carbon-based materials are a good choice to utilize for coatings, due to their excellent chemical stability and physical properties. Carbon coating aims at offering extra ionic diffusion routes and boosting the transport of electrons through the interface on the cathode surface. Meanwhile, carbon coating could not only control the surface chemical stability of cathode materials and their structure change during lithiation/de-lithiation reaction, but also suppress the adverse reactions between cathode and electrolyte, which caused cycling instability. In addition, carbon coating forms a physical covering layer to diminish the corrosion of electrolytes, which raises the specific capacity, strengthens the thermal stability and prolongs the cycle lifetime of the LIB.

The purpose of this paper is to review the recent progress of carbon coatings on improving the performances of the several common cathode materials mentioned above. Importantly, the modification mechanism of carbon coating is also highlighted. Furthermore, typical carbon coating techniques are presented. The impact of carbon layer thickness, carbon content and carbon source on the effect of carbon coating on several cathode materials is also systematically summarized. In addition, this work analyzes the deterioration mechanism of different cathode materials. Finally, some suggestions on developing carbon coating methods that could attain entire and homogeneous carbon members for further research and outlook are presented.

## 2. Impact of Carbon Coating on Cathode

**Figure 1:**
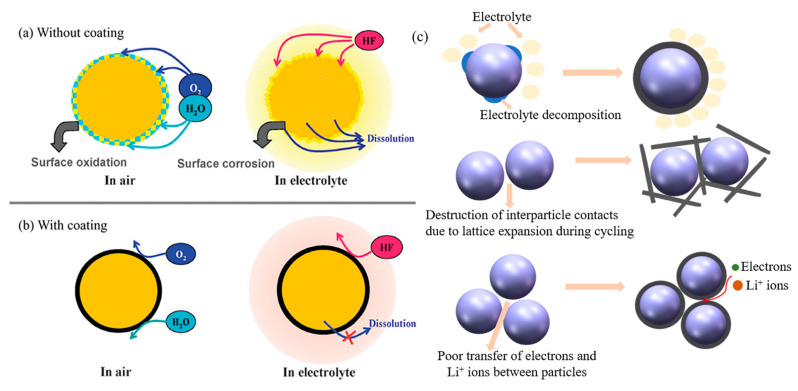
The mechanisms of cathode materials without carbon coating (**a**) and with carbon coating (**b**) (Reprinted with permission from Ref. [17]. Copyright 2012 Royal Society of Chemistry); (**c**) the modification mechanisms of carbon coating on cathode: modifying surface chemistry, enhancing structural stability and improving Li-ions diffusion.

Surface coating is a well-known optimum approach to protect the cathode material and improve the specific capacity, thermal stability and electrochemical stability of LIB. Carbon-based materials such as porous carbon, graphene, graphene oxide, carbon nanotubes (CNTs) and reduced graphene oxide (rGO) are the most popular coating materials due to the following advantages (Figure 1a,b) [15,16]: (1) superior chemical and electrochemical stability. Due to the reaction of electrolyte and electrode, carbon material has strong electrochemical stability with good resistance to acid electrolyte corrosion, which only shows electrochemical activity at very low potential. Additionally, carbon material, which is not easily oxidated, could protect cathode material from oxygen and moisture in the air. (2) Carbon exhibits unique physical properties such as anisotropic conductivity, low density, high mechanical strength and structural flexibility [17,18]. (3) Excellent electrical conductivity. Carbon with good conductivity is important for coating materials. (4) Low cost. The resource of carbon is sufficient and low-cost, and the fabrication is simple. In addition, the layer thickness and the conductivity of carbon coating could be adjusted by the carbon content and preparation condition [19]. With these excellent properties, carbon is one of the preferred materials for surface coating on the cathode of LIB. The carbon coating has the main following mechanisms (as shown in Figure 1c): (1) Modifying surface chemical stability, (2) Enhancing structural stability and (3) improving Li-ions diffusion. These three carbon-coating modification mechanisms influence and promote each other. In the charge and discharge processes, carbon coatings could relieve the dissolution of transition metals and reduce defects on the surface of cathode materials. It benefits the improvement of interfacial chemical stability, improving the uniform diffusion of Li-ions. Besides, the 3D structure provided by the carbon layer could prevent the structure collapse of the cathode materials, decreasing the particles crack caused by the structure change [20]. This results in the mitigation of metal dissolution again, and the promotion of electron transmission through the interface of the cathode material particles. Meanwhile, the 3D carbon network provides extra electron-conducting routes that facilitate the electron transfer at the cathode surface.

### 2.1. Modifying Surface Chemistry

Carbon material with good electrochemical stability serves as a protective barrier to prevent the reaction between cathode material and the electrolyte, and surface oxidation. Besides, through isolating the cathode materials and the electrolyte, residual lithium compounds would be cleared away. On the other hand, the most commonly used lithium salt electrolyte, LiPF_6_, spontaneously decomposed and produced HF (Hydrofluoric Acid) as follows:

$${\mathrm{LiPF}}_{6}\to \mathrm{LiF}+{\mathrm{PF}}_{5}$$

$${\mathrm{PF}}_{5}+H_{2}O\to {\mathrm{POF}}_{3}+2\mathrm{HF}$$

The produced HF by hydrolysis would contribute to the dissolution of transition metals and the corrosion of the surface of active material, resulting in the collapse of the cathode structure. Ultimately, the capacity of LIB declines after long term cycling. Therefore, through carbon coating, the issues of the capacity decline and safety could be relieved effectively.

In addition, the carbon layer separates the active cathode material from the oxygen and moisture in the air. For example, in LiFePO_4_, such olivine structure materials still suffer from the surface oxidation exposed to the air for long time storage, though it possesses the properties of thermal stability and structural stability [21]. Meanwhile, due to the low electronic conductivity, metal oxides such as Al_2_O_3_, CuO and MnO are not suitable for olivine structure material coating. As Pagot et al. demonstrated, the addition of CuO would dilute the cathode active phase, resulting in the weight gain of cathode during the charge/discharge reaction [22]. Although Pagot’s group tried to improve the electronic conductivity of the olivine-structures cathode by doping vanadium, niobium or tantalum high-valence transition metals, the challenge of improving the discharge capacity of the Li-ion battery still needed to be addressed [23]. Therefore, carbon as the chemically stable material could prevent the degradation of the active cathode material in the air, also mitigating the capacity fading in the charge/discharge cycling [17].

### 2.2. Enhancing Structural Stability

With various unique physical features of anisotropic conductivity, low density, high mechanical strength and structural flexibility, carbon coating easily formed by chemical vapor deposition render as a physical protective layer on the surface of the active cathode material. The undesired surface reaction can be suppressed by the coating layer, which enhances the lifetime of LIB and retains the stability of structure [24]. The structural stability is mainly improved by limiting the cathode volume expansion/shrinkage caused by the phase transformation and providing support for the cathode particles with nanoscale structure.

The phase transition occurred in the process of lithiation and de-lithiation, which creates a new substance with another phase structure. In the procedure of phase transformation, the crystal lattice of the cathode would generate anisotropy expansion, resulting in particles crack. This serious volume expansion will greatly affect the cyclic performances and structural stability of cathode materials. The repeated expansion and contraction of the volume leads to electrode differentiation and the separation of the cathode particles. The hollow carbon material can provide a free space for the volume expansion of the cathode material during the lithium process, ensuring the structural stability of the cathode material and the smoothness of the Li-ions and electron transport channel in the cyclic reaction. Moreover, with good elasticity, carbon could be fabricated as a thin shell on the cathode material surface, which can accommodate the change of volume during Li+ insertion and extraction. Zhang et al. [25] mentioned that the nanoparticles encapsulated elastic hollow carbon spheres can not only avoid the cracking and pulverization of the active material, but also work as buffer and container to enhance the cycle performance and volume capacity.

On the other hand, the nano-crystallization of cathode materials is utilized to shorten the diffusion path of Li-ions and enlarge the specific surface area of the material to provide more diffusion routes for the interfacial reaction. Thus, for the nanoscale active material, carbon coating has positive effect on maintaining the nanomorphology. The preparation of a lot of active materials needs to proceed under high temperature (700–1000 °C) to calcine, meeting the requirement of high crystallization. However, the rapid growth and morphology deterioration occur during the process of calcination. The carbon layer can be a solid barrier between active particles to protect them from becoming large particles during calcination [17].

### 2.3. Improving Li-Ions Diffusion

Compared with other coating materials, carbon is an excellent electronic conductor. The lithium diffusion of electrode material is one of the most important factors to determine the performance and capacity of LIB, which is extremely essential for cathode material. The mechanisms of Li-ions’ diffusion include diffusion without vacancy, diffusion with vacancy and phase boundary diffusion [26]. Vacancy diffusion plays an important role in most electrode materials, because of the presence of Li vacancies during the charging process. The lattice change of electrode is small in the process of Li insertion and extraction. The phase boundary diffusion including three typical models of the mosaic model, the shrinking core model, and the domino-cascade model refers that Li-ions diffuse from the surface to the particle center by the interface between the lithiation and de-lithiation phases in the limited solubility [27]. To improve the diffusion of Li-ions, coating with an electronic conductive layer for each nanoparticle, the electronic transport length could shrink effectively to the radius of nanoparticles, forming continuous transport channels in the electrode to allow electrons to pass through the surface of each nanoparticle, which effectively reduces the interface resistance between each of the particles [17]. The Li-ions diffusion limited the rate of Li-ions during intercalation/deintercalation process, and the high resistance of the forming material, which could decline the diffusion. Moreover, working temperature also has an important influence on Li-ions diffusion, and the generated heat in the charge/discharge process increases with the increasing square of internal resistance. Decreasing the particle size of cathode material could effectively reduce the internal resistance, however, the transfer of particles along the interfaces would aggravate the resistance. To reduce the interface resistance, carbon plays an important role in filling the grain boundaries, due to its high electrical conductivity and the ability of transferring Li-ions [27]. The electrons transfer has an impact on the Li-ions migration. The carbon coating layer on the active particles could provide fast electrons and are permeable for Li-ions from the electrolyte.

## 3. Carbon Coating Method

The effect of carbon coating is also significantly affected by the coating techniques. Firstly, different coating methods would lead to the diverse microstructure of coating layer, which results in various ability of Li-ions diffusion through the coating. Furthermore, the coating methods may have impacts on the surface structure of cathode. Therefore, various coating methods have been studied according to different cathode material structures. Meanwhile, the carbon layer prepared by surface coating methods still had problems of inhomogeneity and incomplete cover, the techniques which could fabricate a more uniform and thinner carbon layer are studied. The carbon coating methods can be divided into two categories: wet chemical method and drying coating.

### 3.1. Wet Chemical Methods

The wet chemical methods are the traditional techniques for surface coating on electrode material, which are the most widely used methods in the market production, including hydrothermal/solvothermal, sol-gel, chemical polymerization routes, etc.

#### 3.1.1. Hydrothermal/Solvothermal

In a typical routine, the precursors with solvents are added to an autoclave at a set temperature, and placed the autoclave in an oven for some time. Afterward the reaction solution is cooled to room temperature followed by drying and annealing. For example, Qi et al. [28] reported that using hydrothermal reaction and followed by heat treatment to prepare LiFePO_4_ and LiFePO_4_ microspheres coated with carbon, mixing the sucrose and LiFePO_4_ then transferring the reaction solution into the autoclave for some time followed by cooling to room temperature and drying. The results showed the increasing electrochemical performance and cycling stability. This simple method can reduce the production cost and improve the host structural stability by surface coating. However, it is still a challenge to form uniform coating layer on the electrode surface and form a complete physical protection layer [29].

#### 3.1.2. Sol-Gel Method

Sol-gel method is one of the conventional approaches of coating inorganic materials on the surface of electrode, including two main reactions: (1) hydrolysis of the precursor and (2) polycondensation of the hydrolyzed products to form a polymeric network [30]. Using sol-gel approach to prepare the coating layer on the surface of electrode has the follow advantages: (1) the process can be conducted at a lower temperature which prevents the host structure damage caused by carbon reduction due to high temperature when coating certain electrode materials like NCA or NCM, (2) shorter calcination time and efficient control over stoichiometric-ratio, (3) forming compound with good crystallinity and (4) making uniform particle size distribution and smaller size particle size in the nanoscale. It is an available technique for large-scale production with relative lower production cost and simple operation. However, this method experiences problems involving the low yield, high cost of precursor, heterogeneous and discontinuous carbon coating layer and the generation and change of acidic gases during heat treatment [29].

#### 3.1.3. The Chemical Polymerization Routes

These routes can be divided into two types including in situ and ex situ. The in situ is a preparation method that the precursors which dissolved in the polymeric solution deposited on the surface of host material particles by the chemical reduction, thermolysis, photolysis, etc. In situ method could remain the homogeneous distribution of the polymer matrix and suppress the agglomeration of the host material particles. In the ex-situ process, the host material was prepared by wet chemical method before dispersing into the polymeric matrices [29]. It was believed that ex situ method was the most common and simple approach in the surface coating methods for cathode material, preparing the host material by a wet chemical approach before dispersing it into the polymer matrix. With the relatively high technical maturity, it effectively provides a protective layer against the corrosion of HF and increases interfacial stability between electrode and electrolyte. Besides, ex situ can be used for the large-scale production of surface coating for cathode material, however this method is difficult to achieve uniform distribution of the matrix particles during the dispersion process of polymer matrix [29].

### 3.2. Dry Coating

Recently, due to the cost-effective and environmentally friendly properties, dry coating technique has attracted widespread attention. The larger size particle can be coated mechanically by nanoparticles through dry coating method which forms a core-shell structure. This method includes chemical vapor deposition, atomic layer deposition, physical vapor deposition, etc.

#### 3.2.1. High-Temperature Solid-State Method

High-temperature solid-state method is one of relatively simple dry coating techniques. The carbon-coated cathode materials can be obtained by mixing the cathode powder material and carbon source to get uniform powder, then calcinating at high temperature for several hours. For instance, Cao et al. [31] reported the synthesis route of high temperature solid-state to prepare carbon-coated LiMn_2_O_4_. The products of MnO_2_ nanowire and glucose were put into a Teflon-lined stainless autoclave in the 180 °C, attaining carbon-coated MnO_2_ nanowire. Then, making carbon-coated MnO_2_ nanowire and LiOH anneal at high temperature for several hours in air environment can prepare LiMn_2_O_4_ coated with carbon layer. The previous research indicated the uniform and complete carbon layer could be attained by dry coating, and the electrochemical performance of Li-ion battery can be improved. However, due to the distinctive physical and chemical properties of different coating material, the optimal calcination or annealing temperature depend on the different coating material, which still needs further exploration.

#### 3.2.2. Chemical Vapor Deposition

Chemical vapor deposition (CVD) is one of the most common techniques used to prepare high-quality and high-performance thin film coating on the surface of host materials [32]. The process of CVD mainly involves two steps: (1) exposing the host material to the precursors with volatility, (2) the reaction or decomposition of precursors form a thin film on the substrate surface. For example, Tian et al. [33] coated carbon on the surface of LiFePO_4_ by CVD in a quartz tube. The solid glucose as the carbon source decomposed, forming vapor when the quartz tube was heated up to 550 °C and condensing in the form of small carbon clusters on the surface of LiFePO_4_. It showed that CVD was a novel environmentally friendly and controllable carbon coating method to get more uniform carbon layer, making LiFePO_4_ possess good rate capacity, long cycling lifetime and high-power densities. However, the lower decomposition rate of CVD caused the longer production time, which is not suitable for large-scale production. In addition, more sophisticated facilities are needed in the CVD process which caused higher production cost. Therefore, this technique still needs to be optimized according to different cathode materials to achieve improvement of electrochemical performance of the electrode.

#### 3.2.3. Physical Vapor Deposition

Physical vapor deposition (PVD) is an important method to prepare the protective film with the properties of anti-corrosion and wear resistance [34]. The deposition of material on the substrate is a line-of-site impingement type for PVD, while it is a multi-directional type for CVD [35]. The process of PVD which conducts at the vacuum includes that solid or liquid material transfer to a vapor phase, then the metal vapor congeals and forms a solid and dense film on the surface of the substrate material [34]. Nevertheless, PVD technique generally operates at high temperature and vacuum, which needs a skilled operator. In addition, the process of PVD requires a cooling water system for heat dissipation.

#### 3.2.4. Atomic Layer Deposition

Atomic layer deposition (ALD) is an emerging technique applied for surface modification of cathode material by forming a thin and homogeneous film to prevent the reaction of electrode and electrolyte to improve the electrochemical performance [36]. In general, two or more precursors containing different elements are needed in the ALD process, which are provided one at a time in sequence to proceed surface coating. The precursors are injected into the reaction chamber to synthesize the desired material by chemical surface reaction, which are separated by inert gas purging to prevent gas phase reactions before injecting the next precursor. Its unique self-terminating growth mechanism is in favor of getting conformality and thickness uniformity of the film. The advantages of ALD are as follows: (1) the thickness of coating layer could be controlled by the number of ALD process cycles and the coating deposited has conformality with the substrate surface, which means the shape of coating layer conforms to the substrate surface so that the thickness of coating is uniform, (2) ALD can be applied to deposit a wide range of materials, both conductive and insulating materials surface, (3) the operation temperature is relatively low and (4) the surface coating can effectively reduce the rate of surface reaction and enhance the ionic conductivity of electrode [29]. However, ALD technique involves the complicated chemical reaction procedures, as well as the high cost of requested facilities. In addition, the excess precursors need to be removed when the coating is finished, which make the coating preparation process more complicated.

## 4. Performance of Carbon Coating on Cathode Materials in LIB

Carbon coating sources can be classified into four types: 0-dimensional (0D), 1-dimensional (1D), 2-dimensional (2D) and 3-dimensional (3D) based on the physical morphology structure of carbon. Although the fullerene C_60_ with a 0D structure is not a common carbon coating source, it remains attractive with its excellent properties of higher electron transport and highly conjugated molecular structure. Liu et al. [37] reported a fluorinated fullerene (C_60_F_48_) as a carbon coating source. Cathode materials coated with C_60_F_48_ show significantly enhanced LIB performance due to decreased reaction resistance and boosted diffusion of Li-ions. CNTs are one of the most common 1D structure carbon coating sources with excellent mechanical, electrical and chemical properties. Additionally, CNTs enhance the electrical contact between cathode particles and increase stability against the chemical degradation [27]. Graphene, one of the most common coating materials with a 2D structure has a unique electronic feature, which effectively improves the electrical conductivity. Recently, the 3D mesoporous carbon coating materials have gained attraction due to their high electrical conductivity. Such structured carbon coating materials provide a spider-like network for Li-ions and electrons to diffuse faster, improving the rate capacity of batteries.

### 4.1. Carbon Coating on Olivine Structure Cathode (LiFePO_4_)

LiFePO_4_ with the Olivine structure has a theoretical capacity of approximately 170 mAh g^−1^ and low electrical conductivity of about 10^−9^ to 10^−11^ S cm^−1^ [38]. It exhibited the properties of thermal stability, excellent cycling, low cost, environmental friendliness, and temperature tolerance, making it a promising choice as cathode for powering electric vehicles and consumer electronics. However, Gu et al. [39] illustrated that there are two main factors including the surface amorphization and releasing of oxygen which restrict the further utilization of LiFePO_4_. With the electrochemical performance tests of LiFePO_4_ coated with graphite LIB after more than 3300 cycles, Gu’s group found the presence of an amorphous layer with disordered structure destroyed the fast Li diffusion channels. Meanwhile, the structural amorphization caused the electronic structure change of the Fe-ions. Although LiFePO_4_ has a stable olivine structure with the strong covalent oxygen bonding, the surface of LiFePO_4_ materials affected the capacity and Li-ions transport of the LiFePO_4_ electrode. After repeated cycling, the structure of LiFePO_4_ became amorphous. Meanwhile, the P–O bond strength was weakened, resulting in the release of some oxygen from the surface layer. This is the possible reason for the drop of the Fe valence state in the surface layer. Therefore, the decreasing of capacity and chemical Li-ions diffusion of LiFePO_4_ was caused by the structural and chemical changes of the surface layer.

Carbon coating is an effective strategy to overcome the shortcomings of LiFePO_4_ to suppress crystal growth and decline electrode polarization. The electrochemical performance and electrical conductivity of LiFePO_4_ could be significantly improved by surface modification method with a carbon coating (as summarized in Table 1). It was shown that the common carbon sources used for coating LiFePO_4_ include CNTs, graphene and monosaccharides. The organic pyrolytic carbon was less used to improve the electrochemical performance of LiFePO_4_ because of its insufficient electronic conductivity. Luo et al. [40] stated that dispersing CNTs uniformly coating on the surface of LiFePO_4_ can form a continuous conductive network to reduce electrode polarization and improve cycle capability of cathode, as well as improve the adsorption and immersion of electrolyte to promote the electrode reaction of LIB. Additionally, the carbon coating can act as a buffer layer between active material particles to suppress cracking during reaction. Most notably, the formation of a 3D framework from the 1D CNTs links the active LiFePO_4_ particles, which can promote the rate capacity performance and cycle stability.

**Figure 2:**
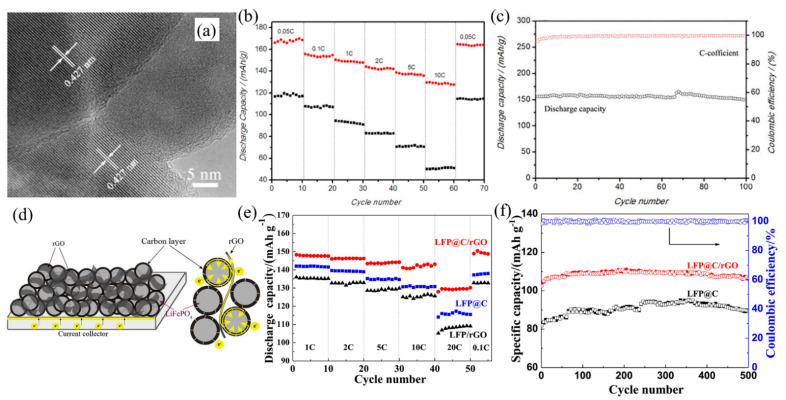
(**a**) HRTEM images of the hydrothermally synthesized LiFePO_4_ with P123. (**b**) Comparison of rate capacity of LiFePO_4_ with carbon nanostructures and LiFePO_4_ with carbon microstructures, and (**c**) cycling performance and Coulombic efficiency of LiFePO_4_ with carbon nanostructures of the cell with the LiFePO_4_ with carbon nanostructures as cathode at the rate of 0.1 C (Reprinted with permission from Ref. [44]. Copyright 2016 Elsevier). (**d**) Schematic illustration of 3D conductive network of rGO and carbon layer in LiFePO_4_ with carbon-rGO composite. (**e**) The rate performance of LiFePO_4_ with carbon, LiFePO_4_-rGO and LiFePO_4_ with carbon-rGO composites. (**f**) The cycle life of LiFePO_4_ with carbon and LiFePO_4_ with carbon-rGO composite at the rate of 1 C (Reprinted with permission from Ref. [46]. Copyright 2018 Elsevier).

Due to the limited improvement in the poor electrical conductivity of LiFePO_4_ coated with graphene by a co-precipitation method, Zhou et al. [43] reported a spray-drying and annealing method to prepare LiFePO_4_ coated with both graphene and glucose-derived amorphous carbon. The LiFePO_4_ particles were coated with a uniform graphene layer with a 3D network, which enabled fast migration of electrons and Li-ions. This result indicated that LiFePO_4_ coated with graphene and carbon has a better rate capability and cycle stability. They explained that the glucose-derived amorphous carbon prevented the stacking of graphene sheets, reducing the anisotropy of electronic migration in the graphene layer and accelerating the Li-ions diffusion through the defects in the graphene sheets. Similarly, Jiang et al. [46] demonstrated the addition of rGO could bridge the carbon-coated LiFePO_4_ nanoparticles (as shown in Figure 2d), improving the homogeneity of the carbon layer and speeding up electrons transfer. The results showed that the rate performance, cycle stability and specific capacity of LiFePO_4_ with carbon-rGO all were get improvements remarkably. In the mutual effect of carbon and rGO, the discharge capacity of LiFePO_4_ with carbon-rGO reached 148.3 mAh g^−1^, which was higher than that of carbon-coated LiFePO_4_ (Figure 2e). In addition, no capacity fading of LiFePO_4_ with carbon-rGO was found at the rate of 10 °C in room temperature after 200 cycles (Figure 2f).

Pratheeksha et al. [48] described a simple and low-cost coating method consisting of a one-step hydrothermal process to prepare in situ carbon coated LiFePO_4_. They also compared the effect on the cathode performance of three different carbon sources, including sucrose, fructose and glucose. The results indicated that this method can form a uniform, thin and highly ordered carbon layer. It was worth noting that using a monosaccharide such as fructose as the carbon source can greatly enhance the electrochemical properties and cycle stability of electrode due to the higher oxygen content and high order of monosaccharide. Controlling the crystal phase of LiFePO_4_ plays an important role in the electrochemical properties. To study the effect of coating layer on the LiFePO_4_ crystal orientation and shape, Wang et al. [45] prepared the LiFePO_4_ coated with glucose as carbon source by a hydrothermal method, exposing the (010) faces in the annealing process. They found that the introduction of carbon content from 1.65 to 6 wt% would change the preferential orientation of the LiFePO_4_ crystal from the (010) plane to the (100) plane.

Minimizing the size of LiFePO_4_ particles is an effective approach to promote the electrochemical performance due to the reduced transport distance of Li-ions and electrons, as well as decreasing the phase transition between LiFePO_4_ particles. Bao et al. [44] reported the plate-like LiFePO_4_ with carbon coating nanostructures (as shown in Figure 2a) could possess better rate performance compared with the carbon-coated plate-like LiFePO_4_ microstructures. The discharge capacity of carbon-coated LiFePO_4_ nanostructure achieved 166 mAh g^−1^, while that of carbon-coated LiFePO_4_ microstructure was 117 mAh g^−1^ (Figure 2b). Moreover, the capacity retention of carbon-coated LiFePO_4_ nanostructure remained 98% (Figure 2c). Additionally, minimizing the particle size of the cathode materials as well as coating the carbon layer can better boost the rate capacity and cycling stability of cathode materials. Qi et al. [28] illustrated the carbon-coated LiFePO_4_ microspheres speeded up the charge transfer and improved electrolyte penetration due to the uniform smaller particles and the addition of carbon source. The LiFePO_4_ with higher carbon content displayed the higher specific discharge capacity of 128.9 mAh g^−1^ and the better cycling stability than that with low carbon content. Nonetheless, it is still a challenge to get a uniform and high-quality graphene coating layer on the surface of LiFePO_4_, limiting the enhancement of conductivity. Fei et al. [41] demonstrated that highly crystalline LiFePO_4_ nanoparticles coated with a homogeneous and continuous graphene nano-shell can be obtained by a solid-state reaction between Fe^0^ wrapped in a graphene nano-shell and LiH_2_PO_4_. It successfully improved the cycle stability and rate capability due to the nanoscale LiFePO_4_ particles and graphene coating layer, which enables the effective transport and diffusion of Li-ions and electrons.

### 4.2. Carbon Coating on Spinels Structure Cathode (LiMn_2_O_4_)

LiMn_2_O_4_ is a typical cathode material with the structure of spinel, which is first commercialized in 1996 [49]. With the theoretical specific capacity of 148 mAh g^−1^, its discharge voltage reaches 4.15 V. Compared to LiCOO_2_, LiMn_2_O_4_ have a longer cycle life in the range of 1000–1500 cycles, lower cost, and higher rate capacity, but lower energy density in the range of 100–140 Wh kg^−1^ [49]. Unfortunately, the transformation between spinel structure (cubic symmetry) and halite structure (square symmetry) would be caused by the Jahn-Teller effect. Thereby this distortion of crystal structure makes the LiMn_2_O_4_ crystal structure suffers from repeated expansion and contraction. This leads to the deformation and the deterioration of the cyclic performance [50]. The capacity loss can also cause by the instability of the λ-MnO_2_ phase, the release of oxygen and self-discharge in the de-lithiation process, leading the solvent oxidation [51]. The loss of capacity and Mn-ions of LiMn_2_O_4_ cathode material at temperature over 50 °C as well as the transformation of LiMn_2_O_4_ phase contribute to the reduced cycle life and exist voltage step [52]. Therefore, the dissolution of Mn in LiMn_2_O_4_ is still a major challenge needed to be solved. A coated carbon layer could reduce the dissolution of Mn effectively, and enhance the electrical conductivity of metal oxides.

**Figure 3:**
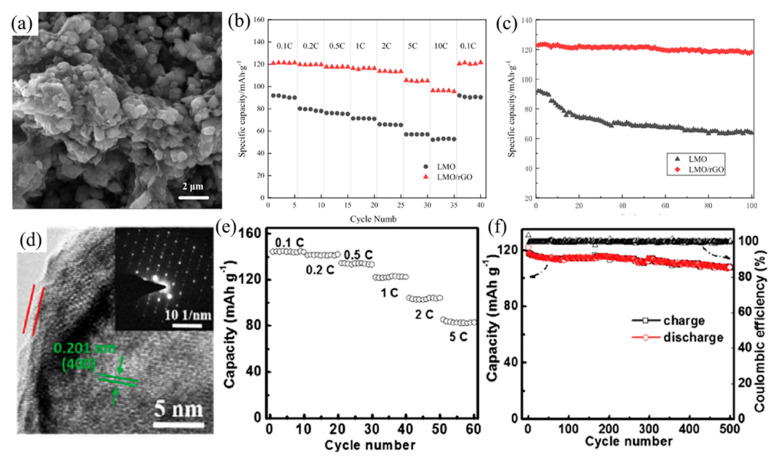
(**a**) The SEM images of LiMn_2_O_4_/rGO; (**b**) discharge rate capability at different current densities; (**c**) cycling performance curve of LiMn_2_O_4_ and LiMn_2_O_4_/rGO at 0.2 C (Reprinted with permission from Ref. [54]. Copyright 2020 Springer Nature). (**d**) The HRTEM diagram of LiMn_2_O_4_ with 10 wt% carbon; (**e**) rate capability under variable current rate; (**f**) cycling performances of LiMn_2_O_4_ with 10 wt% carbon at 1 C (Reprinted with permission from Ref. [31]. Copyright 2018 Elsevier).

The cycling performance and stability of LiMn_2_O_4_-based batteries are improved by coating with CNTs, graphene-based materials, sucrose, etc. As previously mentioned, the carbon coating technique is a favorable solution to the problems associated with LiMn_2_O_4_ including the poor cycling stability, dissolution of Mn^2+^ into the electrolyte and the reaction between the cathode and electrolyte. LiMn_2_O_4_ is a promising cathode material, hence a number of techniques regarding carbon surface modification have been developed (as summarized in Table 2). It is essential that the coating method used does not destroy the structure of LiMn_2_O_4_. Jiang et al. [53] proposed a new cyclohexanone hydrothermal method to synthesize the LiMn_2_O_4_, which can control the particle size in the synthesis process. The results indicated that the CNTs coating layer can be optimized to achieve excellent electrochemical performance without damaging the crystal structure of LiMn_2_O_4_ cathode material. Besides, in order not to destroy the crystal structure of LiMn_2_O_4_, Li et al. [54] also indicated that the host structure was not damaged after coating the cathode material with rGO by the precipitation method (as shown in Figure 3a). They proposed that a binding site could be provided by the oxygen-containing group of graphene oxide for the precursor metal ions, which could reduce the agglomeration of LiMn_2_O_4_ and shorten the diffusion path length of Li-ions, and in favor of the transport of Li-ions. Compared to the LiMn_2_O_4_ without coating rGO, the initial discharge capacity of LiMn_2_O_4_/rGO increased by around 35 mAh g^−1^ (as shown in Figure 3b), and the capacity retention rate was obviously boosted (as shown in Figure 3c).

Zhang et al. [55] proved that LiMn_2_O_4_ coated with CNTs can exhibit better electrochemical performance than LiMn_2_O_4_ without coating because the CNTs coating layer can decrease the contact area between the cathode material and electrolyte. This mitigates the dissolution of manganese, improving the electrochemical cycle performance. Although the discharge capacity of LiMn_2_O_4_ coated with CNTs was lower than that of bare LiMn_2_O_4_ at a low cycle rate, it had better electrochemical performance at a high cycle rate. However, due to the high cost of graphene and CNTs, there is an increasing research focus on carbon coating materials with simple preparation and low production cost. Zhuo et al. [56] reported a graphene-like membrane prepared using liquid polyacrylonitrile as a substitute for graphene. The results showed that the graphene-like membrane with a layered carbon structure will not affect the crystal structure of LiMn_2_O_4_ particles, which improves the discharge capacity and cycling stability. Additionally, dopamine as the carbon source had been reported, and the result illustrated that the carbon coating layer could be fabricated uniformly due to the easy and homogeneous polymerization process from the dopamine solution. However, the oxygen loss in the carbon-coating process led to the deterioration of the phase integrity of LiMn_2_O_4_. Furthermore, LiMn_2_O_4_ with a nanostructure such as nanosheets, nanoparticles, porous structure and nanowires have been investigated. In particular, the 1D nanomaterial has a large surface area, good electron conduction and anisotropic Li-ions diffusion paths, which can shorten the diffusion distance between electrons and Li-ions. Cao et al. [31] illustrated a new approach to prepare carbon-coated single-crystalline LiMn_2_O_4_ nanowire, via agglomeration into the bulk of nanoparticles at high temperature (shown in Figure 3d). This 1D form of material can make Li-ions diffusion paths possess anisotropy, which showed the increasing discharge capacity of 132 mAh g^−1^ at 0.1 C (Figure 3e) and a retention of 90% (Figure 3f) of the initial capacity at 1 C after 500 cycles. Carbon layer can not only inhibit the dissolution of cations to enlarge the capacity of battery, but also sustain the final LiMn_2_O_4_ nanowires morphology during the lithiation/de-lithiation reaction to improve the long-term cycling stability.

### 4.3. Carbon Coating on Layered Oxide Structure Cathode

#### 4.3.1. Carbon Coating on LiCoO_2_ Cathode

LiCoO_2_ is a cathode material with a typical layered oxide structure, with high theoretical capacity of up to about 274 mAh g^−1^. However, the charging voltage of LiCoO_2_-based LIB was limited below 4.25 V and the practical capacity of LiCoO_2_ is only 140 mAh g^−1^, which only accounts for 50% of the total capacity [62]. In the high voltage operation, the failure mechanism of LiCoO_2_ could be classified into three aspects: bulk phase transition, surface degradation and inhomogeneous reaction [63]. The presence of the irreversible phase transition and particle cracks caused by the change of structure and volume in the Li-ions deintercalation/intercalation leads to capacity loss. The previous study indicated that the structure of LiCoO_2_ would change from O_3_ hexagonal phase to H1-3 phase when the cut-off voltage is above 4.45 V [64], resulting in the cracks. Besides, surface degradation can be caused by the impedance growth of LiCoO_2_ electrodes including the continuous formation of the cathode electrolyte interphase, irreversible surface phase transitions, O_2_ loss, and Co dissolution [63]. Based on the inhomogeneous reaction mechanism, the differences of Li diffusion dynamics make the state of charge of different particles or different part of a particle inhomogeneous. Hence, such inhomogeneous distribution of the state of charge caused serious deformation and stress, resulting in the splintering of electrode and particles and the loss of capacity. Furthermore, due to the instability of the LiCoO_2_ surface under the condition of high pressure, it is easy to generate an unstable cathode electrolyte interphase, which is decomposed during cycling process, contributing to the poor cycling performance [63]. Although LIB with LiCoO_2_ has the better stable capacity, it was reported that LiCoO_2_ cathode had safety issues that low thermal stability leading to a runaway reaction and burst into flames [65].

As discussed before, the degradation of LiCoO_2_ is caused by bulk phase transition, surface degradation and inhomogeneous reaction. A lot of studies about LiCoO_2_ coated with carbon from different carbon sources and using different approaches have been reported (as shown in Table 3).

Actually, carbon coating was rarely reported to modify LiCoO_2_ before, because LiCoO_2_ is easily reduced by carbon to CoO or Co_3_O_4_ in the high temperature calcination [67]. Thus, it was reported earlier to coat LiCoO_2_ with carbon black to reduce the amount of conductive agent. However, a carbon black coating layer mainly worked as physical protection without strong contact with LiCoO_2_ [67]. Except the thermal decomposition method, various effective approaches have been introduced, including chemical vapor deposition and mechanical milling. It was suggested by Kwon et al. [68] that the post-treatment technique was better for coating LiCoO_2_ with carbon, and the mechanical milling process possesses the advantages of simple operation, relatively low production cost and providing close contact between LiCoO_2_ and carbon. By contrast, the method of solution mixing is not suitable for the preparation of carbon coatings which would cause an inhomogeneous carbon layer due to the large density difference between LiCoO_2_ and carbon. By testing the LiCoO_2_ coated with platelet-shaped graphite, they demonstrated that the increased specific surface area caused by the reduction of the particle size could provide a larger contact area for the electrode and electrolyte. Additionally, the uniform and thin graphite coating layer allowed electrons and Li-ions to pass through the composite, improving the capacity and cyclic voltammograms. Subsequently, Kwon et al. [69] also indicated that the milling time had important impact on the performances of the carbon coating. Short-time ball-milling can provide a uniform and inmate carbon layer, improving the electrochemical performance by shortening the diffusion length of Li-ions. In comparison, long-time ball-milling increased the number of defects in the structure of carbon, resulting in a thick carbon layer and decreasing the electrochemical performance.

**Figure 4:**
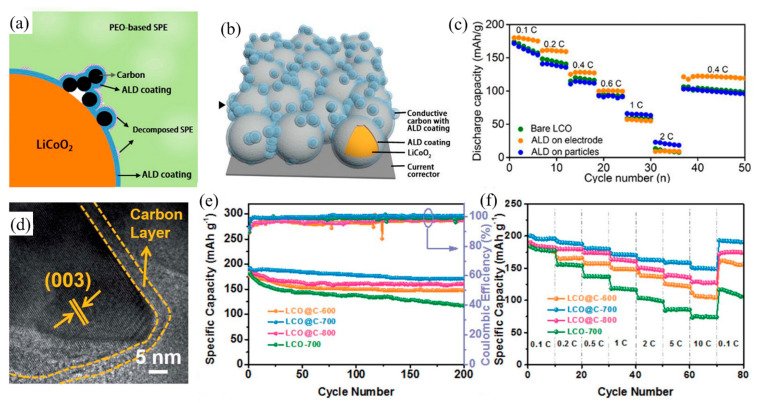
(**a**) The working mechanism of the protected LiCoO_2_ with carbon coating in the ASSLBs after extensive charge/discharge cycles; (**b**) Schematic diagram of the LiCoO_2_ electrode where both LiCoO_2_ and conductive carbon are protected. (**c**) Rate performance of the ASSLBs (Reprinted with permission from Ref. [73]. Copyright 2020 Royal Society of Chemistry). (**d**) HRTEM image of the carbon layer of LiCoO_2_ at a scan rate of 0.1 mV s^1^. (**e**) Cyclability at a current density of 1 C, and (**f**) rate capability at a current density ranging from 0.1 C to 10 C of LiCoO_2_ with carbon coating (Reprinted with permission from Ref. [72]. Copyright 2020 Royal Society of Chemistry).

Wang et al. [70] showed that graphene nanosheets with small size can coat the surface of LiCoO_2_ with a homogenous distribution and without much aggregation, accelerating the transport of electrons and diffusion of Li-ions due to the high electronic conductivity of the graphene network. Nanoscale graphene particles have a high specific surface area and excellent electronic and ionic conductivities. Sun et al. [71] reported that LiCoO_2_ coated with graphene quantum dots by a simple liquid phase approach showed a better cycle stability without the presence of micro-cracks. Luo et al. [74] developed LiCoO_2_ coated with highly aligned CNTs to create a three-dimensional conductive network through a ultrasonication and co-deposition method. The material exhibited good electronic conductivity, high flexibility, high energy density and excellent cycle stability. Liang et al. [73] suggested that atomic layer deposition was an alternative technique to coat carbon on the surface of LiCoO_2_ because atom layer deposition enables accurate control over the thickness of the carbon layer, and it forms a uniform, conformal and thin carbon film (Figure 4a,b). Figure 4a,b showed the schematic diagram of LiCoO_2_ particles and electrode coated by conductive carbon with ALD coating method, respectively. They found that the capacity rate of LiCoO_2_ electrode coated by carbon was remarkably improved at low current density (<0.6 C), while the LiCoO_2_ particles coating achieved the higher capacity at the high current density (Figure 4c). Besides, the most important benefit is that the process uses a relatively low temperature, hence this method would not damage the structure of the carbon and electrode during the preparation. Recently, Lin et al. [72] provided a new strategy of in situ constructing MOF-derived to prepare LiCoO_2_ with carbon-coated core-shell structure by elevated-temperature solid-state annealing. Through several annealing temperature comparison, they found carbon-coated LiCoO_2_ formed in the annealing temperature of 700 °C (Figure 4d) could exhibit the superior electrochemical performance. Figure 4e,f showed the carbon-coated LiCoO_2_ synthesized by the 700 °C annealing temperature has the capacity retention of 89.1% at the current density of 1 C after 200 cycles and the discharge capacity of 193.4 mAh g^−1^ at the current rate of 0.1 C.

#### 4.3.2. Carbon Coating on LiNiO_2_ Cathode

LiNiO_2_ is considered as the alternative of LiCoO_2_ owing to the properties of low cost and a high theoretical capacity of 274 mAh g^−1^ at a reasonable voltage range between 2.6 and 4.2 V [76]. However, LiNiO_2_ could not be commercialized due to its complicated synthesis process, of which the composite of material is difficult to control precisely [77]. In addition, Ni^2+^ ions tend to hinder the diffusion of Li-ions, leading to instability of LiNiO_2_. Firstly, the stoichiometric LiNiO_2_ is difficult to prepare by solid-state reaction method under high temperature due to the high vapor pressure of lithium during the process of calcination, leading to the loss of Li from the host structure, and hence the formation of a non-stoichiometric structure. Such a non-stoichiometric structure makes LiNiO_2_-based LIB have low initial capacity and the serious problem of capacity loss [78]. On the other hand, when charging up to high voltage, the structure of LiNiO_2_ will be changed due to the formation of NiO_2_ phase caused by the irreversible phase transitions. The inactive NiO_2_ phase also reduces the capacity.

**Figure 5:**
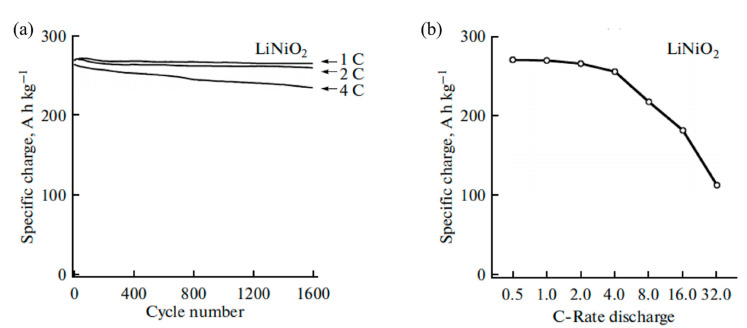
(**a**) Electrochemical specific charge of LiNiO_2_; and (**b**) dependence of the specific charge of LiNiO_2_ (Reprinted with permission from Ref. [79]. Copyright 2015 Springer Nature).

With similar structure, LiNiO_2_ has higher reversible capacity than LiCoO_2_. Due to the difficulty of oxidation of Ni^2+^ to Ni^3+^ during the high temperature synthesis process, it is challenging for large scale preparation. Vandenberg et al. [79] prepared in situ carbon-coated LiNiO_2_ by a microwave-assisted synthesis method, exhibiting an initial specific charge of around 270.5 Ah kg^−1^ at 1 C with an about 98% charge retention at 1 C after 1500 cycles (as shown in Figure 5). However, few studies on carbon-coated LiNiO_2_ have been reported, because coating metallic oxide materials would be more common. Meanwhile, in order to improve the cycling performance and thermal stability of LiNiO_2_, cobalt doping attracts an increasing research attention such as LiNi_0.8_Co_0.2_O_2_ [80].

#### 4.3.3. Carbon Coating on NCM (LiNi_x_Co_y_Mn_1−x−y_O_2_) Cathode

To solve the problem of LiNiO_2_, the strategy of using cobalt, manganese or aluminum as the substitution to decrease the fraction of nickel has been developed, thereby the new composites LiNi_x_Co_y_Mn_1−x−y_O_2_ (NCM) was developed by the Ohzuku et al. in 2001 [81]. With the advantages of relatively low cost, better thermal stability and high capacity, the theoretical capacity of LiNi_0.8_Co_0.1_Mn_0.1_O_2_ (NCM 811) could reach to about 200 mAh g^−1^ [82]. However, NCM exited a major problem of capacity loss due to the degradation of NCM material from nano-scale to micro-scale in the process of discharge and charge [83]. In addition, the oxygen released from NCM with high degree of lithium deintercalation reacts with the organic electrolyte, resulting in the poor thermal stability. In addition, the increasing solid-electrolyte interfacial impedance due to the reduction of Ni^4+^ leaded to poor cycle life [82]. It has been suggested that surface modification is an effective method to improve the performance of NCM. Surface coating can be classified into two types including coating on primary particle level and coating on secondary particle level. It is an effective strategy to prevent the reaction between NCM cathode and electrolyte, also restrains the oxygen evolution of NCM during cycles [81].

To prevent the reaction between NCM cathode and the organic electrolyte, a carbon coating as a physical protection layer and chemical barrier can effectively improve thermal stability of NCM and enhance the electrochemical performance by increasing the Li-ions transport and electronic conductivities. Recently, a lot of different coating methods including chemical vapor deposition, atomic layer deposition and physical vapor deposition, and different coating materials such as graphene, CNTs and sucrose have been investigated, as summarized in Table 4. Chen et al. [84] discussed the issues of carbon coating techniques on Ni-rich cathodes, including that the coating process needed to be conducted without water, and the oxidation of carbon in oxidative environments above 500 °C since the synthesis of NCM generally proceed under an oxygen atmosphere at high temperatures, leading to the oxidization of organic compounds to CO. Therefore, water-soluble carbon sources such as sucrose and glucose are not suitable for coating Ni-rich cathodes at high temperatures and it is still challenging to form a continuous and uniform carbon coating.

For the coating method of NCM cathode, chemical vapor deposition (CVD) and inverse microemulsion routes have been reported. Hou et al. [86] illustrated that the chemical vapor deposition technique could fabricate a more uniform and high-quality carbon coating layer comparing with the traditional coating method of heat evaporation, thus CVD is considered a simple approach to achieve uniform carbon coating. However, the complex process and expensive equipment of these coating methods make them difficult to use in large-scale production. Yang et al. [88] reported a simple method of using highly absorbing activated carbon to absorb the Li-ions and form a uniform carbon layer on the surface of NCM particles. In addition, Al-Shroofy et al. [91] developed a lower cost, high throughput method of solvent-free dry powder coating, compared to the conventional wet slurry-based electrode manufacturing method. Their group made a comparison of the effect of carbon coating by two fabrication methods including dry powder coating and wet-slurry coating, which showed the carbon coating made by dry powder had a higher specific capacity. Moreover, this method also mitigated the fabrication problems of high cost and environmental issues, because dry powder coating can reduce the production cost by simplifying the preparation steps and save drying time, and dry powder coating could relieve the pollution from solvent.

**Figure 6:**
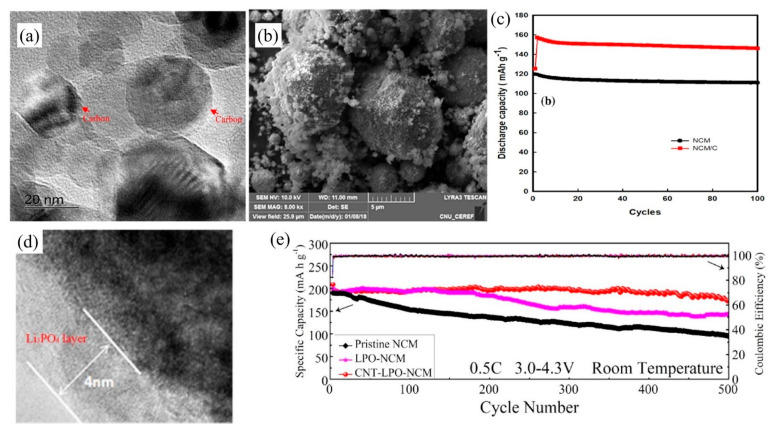
(**a**) TEM images of the NCM coated with carbon layer; (**b**) FE-SEM images of the NCM/C powder; and (**c**) discharge capacity of the NCM/C-based LIB (Reprinted with permission from Ref. [93]. Copyright 2020 Springer Nature). (**d,e**) TEM images of the CNT-LPO-NCM; and cycling performances of pristine NCM, LPO-NCM and CNT-LPO-NCM at 0.5 C separately in the voltage range of 3.0–4.3 V (Reprinted with permission from Ref. [87]. Copyright 2019 American Chemical Society).

For the coating materials of NCM cathode, graphene, CNTs and carbon black are common carbon coating materials. Li et al. [85] reported that unlike coatings of either graphene or CNTs alone, coating both CNTs and graphene enables the formation of a 3D conductive spider web framework in the cathode. CNTs provide an effective link between graphene and NCM to enable fast transportation of electrons and Li-ions, and lower electrode polarization, improving rate capacity and increasing cycling stability. Nguyen et al. [93] illustrated that using soybean oil as the carbon coating source (Figure 6a,b showed the TEM image and FE-SEM images of NCM/C, respectively), exhibited the outstanding advantages of simple fabrication and low cost. As well as, it easily formed a coating layer through an emulsion reaction, and improved the specific capacity and cycling stability. At the same time, they discovered the capacity of NCM/C battery reached to 159 mAh g^−1^ at the 2nd cycle with the gradual wetting of the electrolyte, and maintained 95% of the capacity at 0.1 C after 100 cycles (Figure 6c). Furthermore, Yang et al. [87] demonstrated that a Li_3_PO_4_ (LPO) and CNTs multi-functional coating on the surface of NCM showed high electronic and ionic conductivities, high-rate performance and excellent cycle stability (Figure 6d). Among the pristine NCM, LPO-NCM and CNT-LPO-NCM, the CNT-LPO-NCM showed the most outstanding initial discharge capacity of 202.6 mAh g^−1^ and the best cycling stability with capacity retention of 84.8% at 0.5 C after 500 cycles (Figure 6e). NCM, Li_3_PO_4_, CNTs and the electrolyte form a four-phase cathode electrolyte interface to provide rich electronic pathways and ions channels to improve the electronic conductivity and prevent the corrosion of HF. The thickness of carbon coating layer made a difference in the capacity and cycle performance of NCM. Sim et al. [89] reported the initial discharge capacity of carbon black coated NCM (188.6 mAh g^−1^) was lower than that of the pristine NCM (192.8 mAh g^−1^). The carbon coating layer works as an obstacle because of higher electrode polarization resulting in lower specific capacities while carbon-coated NCM had higher capacity retention and better cyclability.

#### 4.3.4. Carbon Coating on NCA (LiNi_x_Co_y_Al_1-x-y_O_2_) Cathode

NCA (LiNi_x_Co_y_Al_1-x-y_O_2_) is a promising alternative for next generation LIB with high energy density. The advantages of high reversible specific capacity, high cyclic stability, low cost and structure stability because dopant (aluminum) make it successfully applied in electric vehicles including Tesla Mode 3 [94]. LiNi_0.80_Co_0.15_Al_0.05_O_2_ (NCA8115), one of the most common NCA cathode material, could achieve a specific capacity of 265 mAh g^−1^ with high specific energy of about 200 mAh g^−1^, and it was reported the practical capacity could reach about 199 mAh g^−1^, which was higher than that of LiCoO^2^ at an average voltage of 3.7 V [95]. However, it exists the problems of low thermal stability, strict manufacturing condition and residual lithium compounds during synthesis [96]. For the performance deterioration of NCA, there are main four causes including cationic mixing, phase transition, residual lithium compounds, and microcracks, resulting in the presence of deficient cycling performance and thermal stability. Firstly, due to the similar radius between Li^+^ and Ni^+^, it is easy for Ni^+^ to migrate to Li^+^ layer and occupy Li^+^, which makes the thermal instability of the structure of NCA. Moreover, due to the instability of Ni^3+^ and Ni^4+^ at high temperature, the reaction between HF released by the electrolyte and these high oxidation state Ni-ions easily occurs, leading to the structural change and material degradation which affect the capacity and cycle performance of NCA material [97]. Besides, cations mixing makes an impact on the layered-spinel-rock salt phase transition, contributing to the loss of oxygen from the cathodes and the formation of a thick and highly resistive layer improves the transfer impedance. Meanwhile, the capacity of NCA-based LIB decreased with increasing extent of phase transition. Moreover, the fabrication of NCA and the reaction of LiNiO_2_ with H_2_O and CO_2_ from the air both produce the residual lithium compounds on the surface of NCA cathode, leading to the slurry gelation and battery swelling. Furthermore, the microcracks caused by the anisotropic volume changes form the excessive solid electrolyte interface to prevent the diffusion of Li-ions [98]. Therefore, surface coating providing a physical protection is an effective strategy to modify the performance of NCA cathodes, preventing the oxygen evolution and reactions between electrode and electrolyte.

The main problems of NCA include cationic mixing, phase transition and microcracks. Carbon coating is a proven strategy to solve these problems by preventing corrosion by HF and suppressing oxygen volatilization, hence improving the conductivity, enhancing electron transport, reducing polarization and improving the stability of the surface (summarized in Table 5).

Various coating methods have been demonstrated, and the carbon cladding can be classified into three types: core shell structure cladding, ultrathin film cladding and rough cladding [106]. The structure of carbon core shell can indeed improve the capacity and stability of the cathode material; however, the cladding process is complicated and includes two-step co-deposition. In addition, atomic layer deposition is one of the most common methods to prepare a carbon coating layer, which can form a homogeneous and continuous thin carbon film. However, this technique is limited by the target source. Therefore, Zhao et al. [106] proposed a coating method of mixing and rough cladding using a mechanical fusing machine. Although the rough cladding technique is simple and easy to operate, this method still cannot fabricate a continuous carbon layer unlike the other two cladding methods. On the other hand, Yu et al. [100] illustrated that CNTs coated on the surface of NCA by the traditional method of thermal treatment can effectively shorten the channel length for Li-ions transport to improve the electronic conductivity. Carbon thermal reduction would result in damage to the structure of the cathode material during the high temperature heat treatment. Therefore, there is still a need to develop a coating method to form a uniform and continuous carbon layer which can ensure the structural integrity of the cathode material and meet the requirements for mass production. To this end, Park et al. [105] proposed a new carbon coating technique based on the Pickering emulsion processing, which has high scalability, throughput and amenability to recycling. It can greatly decrease the amount of carbon conductive additive and polymer binder, and hence increase the active material percentage and packing density. Furthermore, Park et al. [108] developed a new coating technique, the “collage” technique, which can obtain a uniform carbon coating layer whilst avoiding high-temperature processing and damage to the cathode material. The results showed that this technique can not only provide a sufficiently conductive network, but also increase the electrode density. These new techniques can provide a useful idea for further development of the carbon coating method.

**Figure 7:**
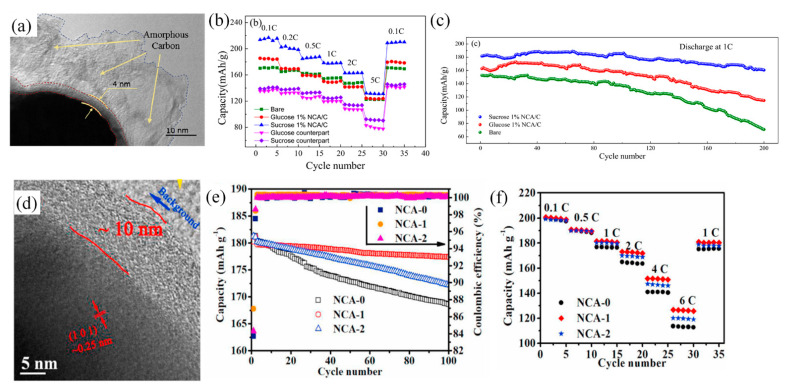
(**a**) TEM images of 1% sucrose-coated NCA; (**b**) rate test of coated and uncoated NCA; and (**c**) cycle performance test of coated and uncoated NCA at 1 C (Reprinted with permission from Ref. [103]. Copyright 2018 Elsevier). (**d**) TEM images of the NCA coated with 8.0 wt% content of the PAN solution. (**e**) Charge/discharge cycle performances of the NCA cathodes; and (**f**) rate cycles of the NCA cathodes (Reprinted with permission from Ref. [107]. Copyright 2021 Elsevier).

For the coating materials of NCA cathode, Visbal et al. [99] proposed a new protective coating layer, diamond-like carbon, to decrease the interface resistance between the electrode and sulfide solid electrolyte. They proved that the diamond-like carbon coating works both as a protection layer and Li-ions mediator. However, they also illustrated the specific capacity of the cathode was affected by the coating layer thickness, because Li-ions need to pass through the diamond-like carbon layer. Gao et al. [101] also indicated that the discharge specific capacity and initial efficiency decreased with increasing thickness of the coating layer, which is caused by the reduction of Ni^3+^ to Ni^2+^, leading to a higher degree of lithium–nickel intercalation in the cathode. In addition, Liu et al. [103] found both of the discharge capacity and the capacity retention of NCA coated with sucrose as carbon source (Figure 7a) were outstanding than that of NCA coated with the same content glucose as carbon source. As Figure 7b shown, the initial discharge specific capacity of sucrose-coated NCA reached over 250 mAh g^−1^ at current density of 0.1 C, as well as the capacity retention of sucrose-coated NCA was 88.3% after 200 cycles at 1 C (Figure 7c). The authors indicated these results attributed to the relatively loose sucrose coating layer with the larger size pores, making the carbon layer has a large specific surface area. It contributed to the movement of Li-ions during the lithiation/de-lithiation process and mitigating the erosion of the electrolyte. To solve the issue of phase transition of NCA, it was reported that an rGO coating layer could suppress the rock salt NiO phase of the NCA nanoparticles and enhance electron transfer and Li-ions diffusion. This fabrication strategy of mechanical wet ball-milling made conformal coating of rGO on the surface of NCA nanoparticles [101]. Generally, it is difficult to form a continuous carbon layer with CNTs and graphene as the carbon source by the conventional methods, and the high cost of these materials makes them only suitable for laboratory research.

On the other hand, in the high temperature process, glucose and sucrose are not suitable as the carbon coating sources due to the generation of H_2_O and CO_2_ from carbon, which would damage the structure of the cathode. Thus, Feng et al. [107] proposed using polyacrylonitrile (PAN) as the carbon coating source, which successfully formed a continuous carbon coating layer on the surface of NCA (Figure 7d). Although, NCA-2 (8.0 wt% content of PAN solution) possessed the highest initial discharge capacity of 181.2 mAh g^−1^, while the NCA-1 (4.0 wt% content of PAN solution) exhibited the highest capacity retention of 98.4% under 1 C current density after 100 cycles (Figure 7e) and the highest capacities at different rates (Figure 7f). The results showed a significant improvement of the electrochemical performance, including the restraint between NCA electrode and environment moisture conditions and the promotion of interfacial dynamics of cathode/electrolyte. The further relationship between carbon content and final electrochemical performance was confirmed.

For further research, it is essential to take the coating material and coating method into consideration. For NCA and NCM, which need to be prepared by heat treatment, polymer as the carbon source is a good choice. Additionally, a growing number of coating methods aimed at simplifying the process and preparing a continuous and uniform carbon coating layer have been reported.

## 5. Limitation of Carbon Coating Method

Although carbon coating on the surface of LIB cathode can effectively enhance the electrochemical performance of battery, several limitations of carbon coating still need to be solved. Actually, many research focused on the effect of carbon coating materials and coating method on the cathode performance, the more comprehensive studies on the influence of different factors such as the suitable coating layer, homogeneity, and the diffusion behavior of electrons are still limited. Therefore, the following aspects should be improved in the further study. (1) The uniformity and integrity of carbon coating layer. It is important to make sure the carbon coating layer completely covers the cathode material particles, and ensures the integrity of carbon coating without structural destruction with increasing cycle times. (2) The surface characteristics of cathode material including pH values and different charged situation of the particle surface. The surface of the cathode material and the coating material should have good compatibility to well modify the cathode material. As Yang et al. mentioned, the addition of p-phenylenediamine (PPD) can remain the reaction pH value to form impurity-free LiMnPO_4_, which shortens the Li-ion diffusion length. In addition, LiMnPO_4_ coating with nitrogen-doped carbon by pyrolysis of the poly (p-phenyleneterephthalamide) (PPTA) and poly (p-phenylene decanamide) (PPDA) showed better conductivity, contact ability and surface capacitance than that of coating with sucrose [109]. (3) Surface modification technologies, such as the combination of coating and doping. Thus far, such studies on combining the surface coating methods have been reported. A recent study involved the strategy of LiNi_0.6_Co_0.2_Mn_0.2_O_2_ (NCM622) with nitrogen-doped carbon coating [110]. It proved that the nitrogen-doped carbon coated NCM622 displayed the improvement of rate performance and capacity retention (92%). The carbon layer with nitrogen doping contributes to achieving the higher degree of cation ordering, moderating the reactions between the cathode and electrolyte as well as increasing electronic conductivity. (4) The selection of carbon content in coating. It is hard to determine the suitable carbon content in coating. For example, the high carbon coating content for LiFePO_4_ will cause the decline of Li^+^ diffusion coefficient, and reduction of specific capacity. By contrast, if the carbon content is insufficient, the positive effect of coating layer on LiFePO_4_ will be indistinctive.

## 6. Conclusions and Outlook

In conclusion, carbon coating greatly affected the performance of LIB through the modification of cathode materials. By modifying surface chemistry, enhancing structural stability and increasing Li-ions diffusion, carbon coating could improve the specific capacity and cycle stability of LIB. Compared with wet chemical methods, drying methods are more prospective in improving the uniformity and coverage of carbon coating layer. By applying deposition techniques, the cathode materials with larger particle size could be coated by the nanoscale carbon particles. In addition, CVD and PVD have become promising carbon coating methods because the traditional carbon coating technique with heat treatment may cause serious structure damage to some cathode materials. Carbon layer thickness, carbon source and coating methods will have an important impact on the performance for different cathode materials. Overall, carbon layer thickness is one of the most important factors to affect the specific capacity and cycling stability of LIB. Meanwhile, compared to other monosaccharides, the specific capacity of cathode coated with carbon from sucrose was more effective. In addition, carbon source from the emerging carbon-based materials coated on the cathode, such as graphene and CNTs, can remarkably improve the specific capacity of LIB. In particular, the specific capacity of NCA coated with sucrose is generally higher than that of several other cathode materials. Moreover, NCM coated with carbon from PVDF/NMP presented high cycling stability. Carbon content is a significant parameter that is difficult to control. Although the specific capacity of high carbon content generally would be higher than that of low carbon content in previous studies, too high carbon content will also reduce the Li-ions diffusion coefficient and results in decreased specific capacity. Therefore, the influences of many factors of carbon coating still need further investigation, such as carbon coating uniformity, surface modification technologies, suitable carbon content and cathode surface characteristics.

In the future, to solve the limitations of carbon coating to improve the performances of LIB, new coating technologies should be developed. The further research in carbon coating can be improved from the following three aspects: (1) developing a more convenient carbon coating method to fabricate homogenous carbon layer, (2) coating with carbon composite materials, and (3) using two-dimensional materials with layered structure to replace carbon materials. Although the wet coating methods are widely applied in laboratories and industrial production, the simpler methods with superiority on coating uniformity are still needed. Carbon composite coating possibly possesses better properties to modify the cathode material and improve the power and energy density of LIB. Furthermore, two-dimensional materials such as transition-metal dichalcogenide (TMDC) with superior excellent properties may replace carbon materials to improve the performance of LIB. This review is expected to inspire the further development of the carbon coating on the performance of LIB.

## Figures and Tables

**Table 1:** Comparison of the cathode performance of LiFePO_4_ with different coating materials and methods.

Carbon Source	Coating Method	Coating (wt%)	Thickness (nm)	LIB Performance		Ref.
				Specific Capacity	Cycling Stability	
Sucrose	Hydrothermal method and heat treatment	15.0	/	128 mAh g^−1^ (0.1 C)	No capacity fading (0.1 C, 50 cycles)	[28]
Carbon nanotubes and glucose	Ultra-fine ball milling and spray-drying	5.0	/	127.1 mAh g^−1^ (10.0 C)	85.3% (10.0 C, 450 cycles)	[40]
Graphene nanosheet	Chemical vapor deposition	5.1	3.66	145 mAh g^−1^ (0.1 C)	95.3% (0.1 C,1000 cycles)	[41]
Graphene and sucrose	Solvothermal, drying and calcination	8.0	5	163.7 mAh g^−1^ (0.1 C) 114 mAh g^−1^ (5.0 C)	97% (0.1 C, 30 cycles)	[42]
Graphene	Spray-drying and annealing process	5.0	2	140 mAh g^−1^ (0.1 C)	95% (20.0 C, 1000 cycles)	[43]
Sucrose	Hydrothermal treatment	/	/	166 mAh g^−1^ (0.05 C)	98% (0.1 C, 100 cycles)	[44]
Glucose	Hydrothermal synthesis and annealing process	1.65	/	162 mAh g^−1^ (0.1 C)	No capacity fading (5.0 C, 50 cycles)	[45]
Graphene oxide and sucrose	Solvothermal method and high temperature solid state reaction	10.0	2–4	148.3 mAh g^−1^ (1.0 C)	No capacity fading (10.0 C, 200 cycles)	[46]
New carbon black and polystyrene	Ball-milling and heat treatment	6.0–8.0	/	160 mAh g^−1^ (0.5 C)	/	[47]
Fructose	Hydrothermal process	8.0	<5	Fructose: 98 mAh g^−1^ (0.1 C) Sucrose: 116 mAh g^−1^ (0.1 C) Glucose: 63 mAh g^−1^ (0.1 C)	/	[48]
Sucrose						
Glucose						

**Table 2:** Comparison of the cathode performance of LiMn_2_O_4_ with different coating materials and methods.

Carbon Source	Coating Method	Coating (wt%)	Thickness (nm)	LIB Performance		Ref.
				Specific Capacity	Cycling Stability	
Glucose	High temperature solid-state method	10.0	3	132 mAh g^−1^ (0.1 C)	90% (1.0 C, 500 cycles)	[31]
Glucose	Hydrothermal method and heat treatment	10.0	1.5	138.5 mAh g^−1^ (0.1 C)	97.76% (0.1 C, 100 cycles)	[53]
Reduced graphene oxide	Ball-milling and calcination	5.0	/	127 mAh g^−1^ (0.1 C)	96.2% (0.2 C, 100 cycles)	[54]
Carbon nanotubes	High temperature solid-state reaction	5.0	/	110.3 mAh g^−1^ (1.0 C)	98% (1.0 C, 20 cycles)	[55]
Liquid-polyacrylonitrile (LPAN) graphene-like membrane	Solid-state ball-milling	20.0	3	131.1 mAh g^−1^ (0.1 C)	96% (0.1 C, 50 cycles)	[56]
Carbon black	Wet slurry and heat treatment	4.0	/	107 mAh g^−1^ (0.5 C)	92.3% (0.5 C, 36 cycles)	[57]
Graphene oxide flakes	Wet chemical and heat treatment	5.0	/	98 mAh g^−1^ (20 mAh g^−1^ current density)	91.2% (20 mAh g^−1^ current density, 100 cycles)	[58]
Polydopamine	Polymerization process of dopamine and heat treatment	0.25 0.65	/	113.3 mAh g^−1^ (70 mAh g^−1^ current density) 93 mAh g^−1^ (70 mAh g^−1^ current density)	51.7% (140 mAh g^−1^ current density, 36 cycles) 73.2% (140 mAh g^−1^ current density, 36 cycles)	[59]
Ethanol	Hydrothermal process and annealing treatment	0.27	/	129.4 mAh g^−1^ (0.5 C)	90% (30.0 C, 1500 cycles)	[60]
Poly (N-vinylformamide)	Mixing in solvent and heat treatment	5.0	2–3	121 mAh g^−1^ (1.0 C)	74% (5.0 C, 1700 cycles)	[61]

**Table 3:** Comparison of the cathode performance of LiCoO_2_ with different coating materials and methods.

Carbon Source	Coating Method	Coating (wt%)	Thickness (nm)	LIB Performance		Ref.
				Specific Capacity	Cycling Stability	
Carbon black	Sol-gel method	1.0	/	145 mAh g^−1^ (1.0 C)	/	[66]
Sucrose	Milling and calcination	5.0	/	130 mAh g^−1^ (0.1 C)	/	[67]
Plated-shape graphite	Ball-milling and drying	20.0	/	80 mAh g^−1^ (0.1 C)	/	[68]
Graphite	Milling and drying	10.0	/	220 mAh g^−1^ (0.1 C)	/	[69]
Graphene nanosheet	Dispersing in solution and evaporation	2.1	/	180.8 mAh g^−1^ (0.1 C)	88.5% (0.1 C, 100 cycles)	[70]
Graphene quantum dots	Liquid phase method and filtrating and drying	1.0	10	182.7 mAh g^−1^ (0.1 C)	82.8% (0.5 C, 100 cycles)	[71]
MOF-derived carbon	High temperature solid-state method	14.03	5	193.4 mAh g^−1^ (0.1 C)	89.1% (0.1 C, 200 cycles)	[72]
Carbon black	Mixing solvent and drying	6.0	10	170–177 mAh g^−1^ (0.1 C)	60.3% (0.1 C, 100 cycles)	[73]
Super-aligned Carbon nanotubes	Ultrasonication and co-deposition technique	5.0	20	151.4 mAh g^−1^ (0.1 C)	98.4% (0.1 C, 50 cycles)	[74]
Carbon black	Pyrolysis of resorcinol	0.88	2	147 mAh g^−1^ (0.3 C)	/	[75]

**Table 4:** Comparison of the cathode performance of NCM with different coating materials and methods.

Carbon Source	Coating Method	Coating (wt%)	Thickness (nm)	LIB Performance		Ref.
				Specific Capacity	Cycling Stability	
Polymers	Chemical wetting method and heat treatment	0.39	4	191 mAh g^−1^ (0.5 C)	98.74% (0.2 C, 100 cycles)	[84]
Carbon nanotubes and graphene	Wet chemical method	10.0	/	187 mAh g^−1^ (0.5 C)	93.8% (1.0 C, 50 cycles)	[85]
Sucrose	Chemical vapor deposition	2.5	6	218.2 mAh g^−1^ (0.1 C)	94.78% (0.1 C, 100 cycles)	[86]
Carbon nanotubes	Wet chemical method	0.01	4	202.6 mAh g^−1^ (0.5 C)	84.8% (0.5 C, 500 cycles)	[87]
Active carbon	Sol-gel route	4.1	10	191.2 mAh g^−1^ (0.5 C)	90.3% (1.0 C, 100 cycles)	[88]
Super-P carbon black	RAM (resonant acoustic mixer) and heat treatment	0.5	0.89–1.23	188.6 mAh g^−1^ (0.5 C)	87.8% (0.5 C, 80 cycles)	[89]
Single-walled carbon nanotubes	Chemical wetting method and heat treatment	5.0	8	160 mAh g^−1^ (0.5 C) 130 mAh g^−1^ (5.0 C)	92% (5.0 C, 500 cycles)	[90]
Carbon black	Electrostatic spraying	1.0	/	156 mAh g^−1^ (0.2 C)	80% (0.2 C, 300 cycles)	[91]
Graphene ball	Chemical vapor deposition and wet slurry method	1.0	5	191.6 mAh g^−1^ (0.1 C)	97.3% (1.0 C, 100 cycles)	[92]
Soybean oil	Solid-state method	/	5	159 mAh g^−1^	95% (100 cycles)	[93]

**Table 5:** Comparison of the cathode performance of NCA with different coating materials and methods.

Carbon Source	Coating Method	Coating (wt%)	Thickness (nm)	LIB Performance		Ref.
				Specific Capacity	Cycling Stability	
Diamond-like carbon	Chemical vapor deposition method	5.0	4.3	120.7 mAh g^−1^ (0.05 C)	90% (0.1 C, 100 cycles)	[99]
Multi-walled carbon	high-powder ultrasonic stirring	0.5	/	205.6 mAh g^−1^ (0.1 C)	91.7% (2.0 C, 800 cycles)	[100]
Aniline and phytic	chemical wetting and heat treatment	1.0	8	190 mAh g^−1^ (1.0 C)	90.7% (1.0 C, 200 cycles)	[101]
Reduced graphene oxide	Mechanical wet ball-milling method	1.0	3.9	196 mAh g^−1^ (0.2 C)	91.7% (1.0 C, 100 cycles)	[102]
Sucrose	Chemical wet and heat treatment	1.0	4	250 mAh g^−1^ (0.1 C)	88.3% (1.0 C, 200 cycles)	[103]
Glucose		1.0	3	225 mAh g^−1^ (0.1 C)	70.4% (1.0 C, 200 cycles)	
Graphene	Wet slurry and heat treatment	4.5	<20	190 mAh g^−1^ (0.1 C)	60.5% (1.0 C, 200 cycles)	[104]
Graphene	Pickering emulsion process	0.5	<10	191 mAh g^−1^ (0.1 C)	70% (1.0 C, 250 cycles)	[105]
Graphite sheets	Mixing and cladding process by a mechanical fusing machine	8	/	181 mAh g^−1^ (0.2 C)	85% (0.5 C, 400 cycles)	[106]
Polyacryloni-trile (PAN)	Chemical wet and high temperature heat treatment	4	5	180.2 mAh g^−1^ (1.0 C)	98.4% (1.0 C, 100 cycles)	[107]
Graphene	Sonication and “collage” technique	1.0	3.1	208 mAh g^−1^ (0.1 C)	72% (0.5 C,100 cycles)	[108]

## Data Availability

Not applicable.
